# Holding a stick at both ends: on faces and expertise

**DOI:** 10.3389/fnhum.2014.00442

**Published:** 2014-06-20

**Authors:** Assaf Harel, Dwight J. Kravitz, Chris I. Baker

**Affiliations:** ^1^Laboratory of Brain and Cognition, National Institute of Mental Health, National Institutes of HealthBethesda, MD, USA; ^2^Department of Psychology, The George Washington UniversityWashington, DC, USA

**Keywords:** expertise, perceptual expertise, object recognition, visual perception, fMRI, review, visual cortex

Ever since Diamond and Carey's (1986) seminal work, object expertise has often been viewed through the prism of face perception (for a thorough discussion, see Tanaka and Gauthier, 1997; Sheinberg and Tarr, 2010). According to Wong and Wong (2014, W&W), however, this emphasis has simply been a response to the question of modularity of face perception, and has not been about expertise in and of itself. It is precisely this conflation of questions of expertise and modularity, the consequent focus on FFA, and the detrimental effect this had on the field of object expertise research that we discussed as part of our original review (Harel et al., 2013).

We fully acknowledge that some recent works on visual expertise—particularly outside the domain of real world object recognition (the focus of our article)—have started to discuss object expertise beyond sensory cortex (e.g., Wong and Gauthier, 2010; Wong et al., 2012). However, at the same time, other high-profile works continue to focus on expertise solely in the context of FFA and face-selectivity (McGugin et al., 2012, 2014), arguing that their results are inconsistent with the notion that “learning effects are distributed throughout cortex with no relation to face selectivity” (McGugin et al., 2012, p. 17067).

Focusing on discrete regions when the question is modularity, but focusing on distributed effects when the question is expertise itself, comes across as holding the stick at both ends and leads to the widespread misconception that FFA plays a privileged role in expertise. Of course, one can show that expertise effects occur within FFA while simultaneously acknowledging the widespread effects of expertise across the cortex. However, the significance of the former result to the understanding of object expertise is greatly reduced by the latter. Put simply, the more distributed expertise effects are, the less significant is the role of one particular region for our understanding of the general mechanisms of object expertise. Take for example, the widespread effects of car expertise, which includes even early visual cortex (Harel et al., 2013, Figure 2). Thus, a continued focus on the relationship between expertise and face processing detracts from the study of the general principles underlying real-world object expertise.

Beyond the issue of modularity, W&W suggest we mischaracterized prior research by stating it often emphasized expertise as an automatic, stimulus-driven skill, with little impact of attention, task and high-level cognitive factors. However, we found this criticism rather surprising given (i) the extensive discussion of automaticity and interference in the expertise literature with many studies suggesting that processing becomes more automatic with expertise (Tarr and Gauthier, 2000; Gauthier and Tarr, 2002; McCandliss et al., 2003; McGugin et al., 2011; Richler et al., 2011), and (ii) recent work explicitly testing the hypothesis that car expertise effects are invariant to modulations of attention or clutter (McGugin et al., 2014). In their response W&W suggest that experts “*tend* to automatically process their objects of expertise in a certain way” but those processes can be “*overridden* by higher-level cognitive processing.” It is unclear how an automatic process can be sometimes engaged and occasionally overridden. This comes across as another instance of holding the stick at both ends.

Despite the points of contention we have highlighted here, we are encouraged that W&W fully agree with the distributed interactive view of visual expertise we discussed (Harel et al., 2010, 2013). We are certain that future research fully focused on addressing the distributed and highly interactive nature of visual expertise will provide new insights into the cortical mechanisms underlying real world object expertise.

## Conflict of interest statement

The authors declare that the research was conducted in the absence of any commercial or financial relationships that could be construed as a potential conflict of interest.
